# Machine Learning Detection of Peritoneal Entry from Vibroacoustic Signals During Veress Needle Insertion

**DOI:** 10.3390/diagnostics16203297

**Published:** 2026-10-10

**Authors:** Moritz Spiller, Mohammad Rezapourian, Axel Boese, Alfredo Illanes, Thomas Neumuth, Nazila Esmaeili

**Affiliations:** 1 Innovation Center Computer Assisted Surgery (ICCAS), University of Leipzig, Semmelweissstr. 14, 04103 Leipzig, Germany; 2 Department of Neuroradiology, Medical Faculty, Otto-von-Guericke University, Leipziger Str. 44, 39120 Magdeburg, Germany; mohammad.rezapourian@med.ovgu.de; 3 Faculty of Computer Science and Research Campus STIMULATE, Otto-von-Guericke University, Universitätsplatz 2, 39106 Magdeburg, Germany; alfredo.illanes@ovgu.de; 4 Institute for Medical Technology, “Medical Technical Systems”, Otto-von-Guericke University, Universitätsplatz 2, 39106 Magdeburg, Germany; 5 Center for Digital Surgery, Department of General, Visceral, and Pediatric Surgery, University Medical Center Goettingen, Robert-Koch-Straße 40, 37075 Göttingen, Germany; nazila.esmaeili@med.uni-goettingen.de

**Keywords:** vibroacoustic sensing, laparoscopic access, Veress needle, peritoneal puncture, convolutional neural network, surgical guidance

## Abstract

**Background/Objectives**: Establishing access is a hazardous step of laparoscopic surgery, and the widely used Veress needle offers only subjective tactile and auditory cues for peritoneal entry. Proximal vibroacoustic (VA) sensing captures needle–tissue interaction non-invasively and is known to contain a characteristic peritoneal puncture signature, which could help surgeons to precisely identify peritoneal entry. However, the VA signature has been evaluated only by retrospective, video-anchored and manual signal analysis. We investigate whether peritoneal entry can instead be detected automatically. **Methods**: Cavity entry is framed as a binary classification of VA signal segments, and four model families are benchmarked under a single unified protocol: a one-dimensional convolutional neural network (1D CNN), a multilayer perceptron, a bidirectional long short-term memory network (LSTM), and a support vector machine (SVM) with a radial basis function kernel. All models were trained, selected, and threshold-tuned on a synthetic abdominal phantom (996 segments) and tested once on an independent porcine cadaver dataset (154 cavity, 462 non-cavity signals) so that every test result measures generalisation across a phantom-to-porcine domain shift. **Results**: The 1D CNN performed the best (accuracy 0.869, AUROC 0.904, macro F1 0.833, cavity recall 0.818), closely followed by the SVM (AUROC 0.900, AUPRC 0.878). **Conclusions**: These results establish the feasibility of automatically detecting peritoneal entry across a phantom-to-porcine domain shift and extend the descriptive cadaveric characterisation of the puncture signature toward the automated machine learning-based detection of peritoneal entry in laparoscopic access with a Veress needle.

## 1. Introduction

Minimally invasive surgery offers shorter recovery, less postoperative pain, and reduced blood loss than open surgery. However, setting up laparoscopic access remains a risky procedure. Between 30% and 50% of all laparoscopic complications occur during the creation of the access [1,2,3], and many of the resulting vascular and visceral injuries are not recognised intraoperatively, with reported mortality rates of 5–15% [4,5,6]. Under-reporting suggests that the true incidence is likely higher [7,8]. The Veress needle remains the most widely used closed-entry instrument, yet its safety depends almost entirely on the surgeon’s tactile perception and on the audible click of the spring mechanism, which is an inherently subjective cue. In one survey, 36% of surgeons felt unsafe using it, and 55% reported that the spring mechanism does not clearly indicate peritoneal entry [9], and systematic reviews identify no entry technique as the unequivocally safest [3,10,11].

Numerous guidance-enhanced instruments have been proposed, yet none have displaced standard practice. These include optical trocars [12,13,14]; modified Veress needles with visual indicators, integrated cameras, or optical coherence tomography probes [15,16]; sensor-augmented trocars and needles based on force, impedance, or accelerometer sensing [17,18,19,20,21]; and mechanical adjuncts and technique modifications [22,23,24]. These solutions are typically proprietary and single-use and require sensing elements within the instrument shaft or tip, so conventional Veress needles and trocars continue to dominate practice [25,26,27].

The vibroacoustic (VA) sensing of the tool–tissue interaction offers a different route. Vibrations generated at the interaction site propagate along the instrument to its proximal end, where they can be captured non-invasively, without modifying the instrument or placing any active component inside the patient [28,29]. The technique discriminates tissue-layer transitions [28] and has been applied to robotic palpation, computerised tissue differentiation, and acoustic sensing in laparoscopic surgery [30,31,32]. For the Veress needle specifically, VA sensing has been studied in ex vivo tissue and animal cadavers [33,34] and recently in a human body donor [35], reproducibly identifying a characteristic sequence of puncture phases in which cavity entry appears as a high-energy, weakly damped transient. These studies identify the event, however, by retrospective manual annotation anchored to a synchronised video, and whether it can be recognised automatically, and robustly enough for real-time guidance, remains an open question.

Within the surgical and needle-based domain, only a handful of studies have applied machine learning to VA signals, and each targets a different problem than transient event detection, namely tissue type during needle insertion into a gelatin-embedded phantom [36,37], material density and structure [38], osteoarthritic versus healthy cartilage in vibroarthrographic knee signals [39], tissue temperature [40], and object recognition from non-contact VA signals [41]. Across these works, the data originate from simple phantoms or non-biological objects, and alternative model families are rarely benchmarked against one another. Beyond VA sensing, 1D-CNNs, LSTMs, and convolutional–recurrent hybrids perform strongly on phonocardiogram, electrocardiogram (ECG), electroencephalogram (EEG), and gait time-series [42,43,44,45,46], but none of these addresses the detection of a transient puncture event in VA signals from a surgical instrument–tissue interaction.

To the best of our knowledge, no study has applied machine learning or deep learning classifiers to VA signals from needle–tissue interactions in porcine cadavers. The present study addresses this gap. It asks whether the peritoneal entry event during Veress needle insertion can be distinguished automatically from other tool–tissue interactions in the proximally acquired VA signal, framing the task as a binary classification of cavity versus non-cavity events. Four representative classifiers are compared under a single unified protocol, namely a one-dimensional convolutional neural network (1D CNN) [47,48], a long short-term memory network (LSTM) [49], a multilayer perceptron (MLP) [50,51], and a support vector machine (SVM) with a radial basis function (RBF) kernel [52]. All model development, selection, and decision threshold tuning are performed on a controlled synthetic abdominal phantom, and the models are tested a single time on an independent porcine cadaver dataset so that every reported test result measures generalisation across a phantom-to-porcine domain shift. The best-performing model is further analysed through threshold sensitivity and ablation studies, as a step toward a surgeon-independent indication of peritoneal entry that can be integrated with standard Veress needles and existing surgical workflows.

## 2. Materials and Methods

### 2.1. Dataset Description

**Figure 1:**
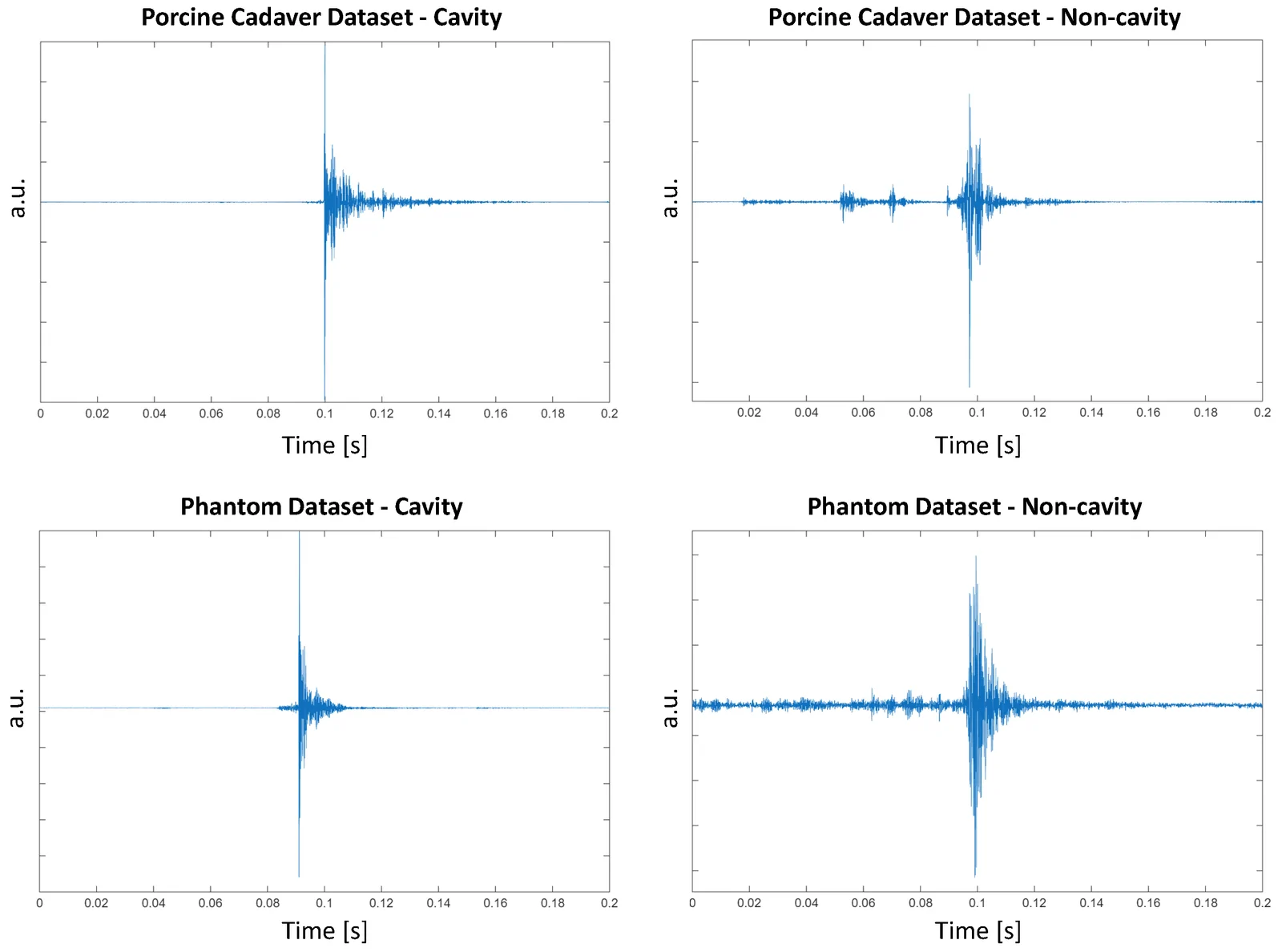
Exemplary VA signal segments of the cavity and non-cavity classes in the porcine cadaver dataset (**top**) and in the phantom dataset (**bottom**). Amplitudes are given in arbitrary units (a.u.).

The datasets consist of VA signals recorded during Veress needle insertion in an artificial phantom and in porcine cadavers. The phantom dataset was used for model development, training, and validation, while the porcine cadaver dataset was reserved exclusively for the final test. Table 1 summarises the structure of both datasets, and Figure 1 shows exemplary VA signals of both classes in each dataset.

The clinical objective is to determine whether a recorded VA segment corresponds to a successful entry of the needle into the abdominal cavity. This is framed as a binary classification problem, with cavity puncture events as the positive class and non-cavity events as the negative class. Depending on the dataset partition, the non-cavity class comprises fascial resistance events together with artefactual or unsuccessful insertions.

#### 2.1.1. Sensing System and Data Acquisition

Both experiments used the same sensing system. The sensing unit comprises a custom printed circuit board (PCB) with buttons, LEDs, and a sensing element, mounted on a Raspberry Pi Zero W (Raspberry Pi Foundation, Cambridge, UK). Its housing terminates in a male Luer connector that mates with the female Luer of a 150 mm single-use Veress needle (Applied Medical, Rancho Santa Margarita, CA, USA), leaving the needle itself unmodified. The experimental protocol and the sensing device are described in more detail in [34]. Signals were acquired at a sampling frequency of 16 kHz and stored in WAV format on an off-the-shelf notebook, a Lenovo T460 (Lenovo Group Limited, Beijing, China), that was connected via Wi-Fi to the sensing unit. For annotation, the recordings were read and stored as MATLAB Version R2026a (The MathWorks Inc., Natick, MA, USA) .mat files, which then served as the input to the classification pipeline.

#### 2.1.2. Phantom Dataset

Model development, training, and validation were carried out on VA signals acquired from a synthetic abdominal phantom. The phantom consisted of a large skin suture pad measuring 13 × 15 × 1 cm (Erler-Zimmer Medical GmbH, Lauf, Germany) placed on a commercially available iron-on interfacing. The suture pad is composed of two layers that are glued together, with the first layer replicating fat and the second layer replicating muscle. The transition between those layers replicates the fascial events. The interfacing beneath the pad represented the peritoneum so that the needle passed through fat, muscle, and the peritoneum in the same order as in the abdominal wall. The phantom was not designed to reproduce accurate tissue properties. Its main objective was to replicate the transitions between tissue layers and the associated friction.

From the phantom recordings, annotated VA segments were extracted. This yielded 996 labelled segments, comprising 380 cavity punctures, 362 fascial resistance events, and 254 artefacts. For the binary task, the cavity punctures formed the positive class, while the fascia and artefact segments were merged into the negative class, resulting in 380 positive and 616 negative samples.

#### 2.1.3. Porcine Cadaver Dataset

An independent test set was constructed from Veress needle insertions in porcine cadaver models and reserved exclusively for final evaluation. It was never used for training, model selection, or hyperparameter tuning and contained 154 cavity and 462 non-cavity segments acquired from eight fresh porcine cadavers under the same experimental setup as the phantom. The needle was inserted as in a normal surgical procedure, in which the Veress needle is placed first, and insufflation is performed afterwards, so the peritoneal cavity was not insufflated during the recordings. The porcine data are considerably more demanding than the phantom data, introducing greater biological variability, more realistic tissue heterogeneity, and signal characteristics that the phantom does not fully capture. Performance on this set therefore offers a more clinically meaningful estimate of generalisation.

#### 2.1.4. Annotation

The cavity and non-cavity labels originate from a manual annotation stage and were read directly by the data-loading pipeline rather than re-derived at load time. Annotation was performed by an experienced professional familiar with medical data processing, following the procedure established in the prior study [35]. For the porcine dataset, cavity and non-cavity segments were curated into separate labelled sets during annotation so that the non-cavity set corresponds exactly to the segments identified as non-cavity at that stage. No segment was relabelled, and no automatic or threshold-based criterion was used to derive class membership.

### 2.2. Signal Representation

The VA signals had undergone two preprocessing steps during data preparation. First, the DC offset was removed. Second, background noise was attenuated by spectral gating, in which a frequency-dependent noise threshold estimated from the signal-to-noise ratio was used to build a mask that suppressed components falling below the threshold [53]. Beyond these steps, no further signal-level preprocessing was applied. The signals were not additionally filtered, detrended, normalised, resampled, transformed into the frequency domain, or otherwise enhanced before classification. This allowed us to test whether the discriminatory information for cavity entry can be learned directly from the measured waveform morphology.

The .mat files were read with SciPy’s loadmat function and the amplitude vectors converted into numerical arrays. For the CNN and the neural baselines, each segment was represented as a one-dimensional signal tensor with a single input channel so that every input was treated as a temporal VA waveform rather than a handcrafted feature vector. The same representation was flattened for the non-neural baseline to satisfy its input format.

All models operate on fixed-length inputs of 3201 amplitude samples, the native segment length produced by the acquisition and segmentation protocol. At 16 kHz this corresponds to roughly 200 ms, twice the duration of a puncture event, which lasts on the order of 100 ms [34,35]. A window of about twice the event length ensures that the full transient and its surrounding context are captured even when the event is not centred within the segment. In the phantom dataset, 927 of the 996 segments were natively 3201 samples long, while the remaining 69 were cropped to this length from longer recordings. Every porcine test segment was natively 3201 samples long and required neither cropping nor padding. No segment was shorter than 3201 samples, so zero-padding was never applied, and no amplitude values were altered.

Working directly on the time-domain waveform, rather than on handcrafted features or frequency-domain representations, preserves the transient structure of the puncture event, including the abrupt mechanical changes that further processing can attenuate. This is particularly relevant for Veress needle detection, where the discriminative pattern may be brief, localised, and embedded within a longer VA response.

### 2.3. Training and Validation Strategy

The phantom dataset supported both training and validation, with a split fixed before any training began so that validation samples never influenced optimisation. The validation set was used for model selection, early stopping, and threshold selection, while the porcine dataset was held out entirely and examined only once, after the final configuration and operating threshold had been determined. The phantom data were used only for training and validation and the porcine data only for testing, because the porcine dataset is comparatively small and, on its own, insufficient to train deep models reliably. Pooling the two acquisition domains was also avoided, since it would risk introducing bias.

The 996 phantom segments were partitioned once into 790 training segments (304 cavity, 486 non-cavity) and 206 validation segments (76 cavity, 130 non-cavity), an 80/20 split stratified by class. The partition was group-aware, so all segments from a given insertion event were assigned entirely to one partition, and no event contributed to both. It was generated once, stored, and reused unchanged for every model so that all architectures were trained and selected on identical samples.

Because the task is naturally imbalanced, the training procedure was designed to limit bias toward the majority non-cavity class through weighted binary cross-entropy and positive-class oversampling. The positive-class weight was set to the ratio of negative to positive training samples,

(1) $$w=\frac{N^{-}}{N^{+}},$$

where $N^{-}$ and $N^{+}$ denote the numbers of non-cavity and cavity training samples. The corresponding weighted loss was

(2) L=−wylog(p)−(1−y)log(1−p),

where *y* is the ground-truth label and *p* the predicted probability of cavity entry.

Augmentation was applied during training only and never modified the stored dataset or the validation and test signals. It comprised additive Gaussian noise, random temporal shifting, amplitude scaling, and random temporal masking, chosen to emulate sensor noise, small differences in insertion timing, variability in contact dynamics, and transient artefacts. Because the stored data remained unchanged and all validation and test signals were evaluated without augmentation, the procedure improved robustness while preserving the integrity of the evaluation protocol.

### 2.4. Evaluated Architectures

Four architectures spanning classical and neural approaches were evaluated under a unified protocol to determine which best suits the task. The 1D CNN learns local and multiscale temporal patterns in the waveform, the MLP performs the non-convolutional neural classification of the flattened waveform, the LSTM represents recurrent sequence modelling, and the SVM provides a classical nonlinear baseline.

#### 2.4.1. 1D CNN

The one-dimensional CNN opens with a convolutional stem of two layers with relatively large temporal kernels, providing a broad local receptive field for abrupt amplitude changes and short-duration waveform structures, followed by max pooling. The main body comprises residual convolutional stages with batch normalisation, SiLU activation, and skip connections that ease gradient propagation and support deeper feature extraction. The channel dimension grows across stages, building representations from low-level waveform edges to higher-level puncture signatures. Dilated convolutions in the later layers enlarge the effective receptive field with few additional parameters, letting the model integrate information over wider regions while remaining sensitive to local transients. Subsequently, global average pooling aggregates the temporal feature map into a fixed-dimensional representation, which is processed by a fully connected classification head incorporating dropout regularisation and a terminal sigmoid activation to yield the estimated probability of cavity entry.

#### 2.4.2. Multilayer Perceptron

The MLP receives each VA signal as a flattened vector and contains two fully connected hidden layers with 256 and 64 units. Each hidden layer is followed by batch normalisation, a ReLU activation, and dropout with probability 0.4, and a final linear layer maps the 64-dimensional representation to a single logit, converted to a probability by a sigmoid at evaluation. Because the fully connected layers combine information from all positions, the model can learn global nonlinear relationships between amplitude values, but it has no explicit bias for temporal locality or translation tolerance. Similar puncture-related features at different positions must therefore be represented through separate weight combinations rather than detected by a shared filter. The MLP was included to test whether the VA signal carries discriminative information recoverable through global nonlinear combinations of its samples, independent of local temporal morphology.

#### 2.4.3. LSTM

The recurrent baseline is a single-layer bidirectional LSTM that treats each VA signal as an ordered sequence of amplitude samples with one feature per time step. Each direction uses 64 hidden units, and the forward and backward passes are combined into 128 features per position. The gated cell state allows relevant characteristics to be retained across many time steps while less informative variations are suppressed, which can in principle relate puncture-associated transients to patterns elsewhere in the waveform. The bidirectional output at the final position passes to a head that reduces the 128 features to 32 units, applies ReLU and dropout with probability 0.3, and maps the result to a logit converted by a sigmoid. Unlike the MLP, the LSTM preserves sequential ordering, and unlike the CNN it does not learn translation-tolerant local filters, so its inclusion tests whether recurrent modelling offers an advantage for this task.

#### 2.4.4. SVM with RBF

The classical baseline is a support vector machine with a radial basis function (RBF) kernel, applied to the flattened waveform with balanced class weights. Each signal was converted into a fixed-length vector of ordered amplitude samples, with no convolutional, recurrent, or handcrafted feature stage, so the classifier operated directly in the high-dimensional waveform space. For two waveform vectors $x_{i}$ and $x_{j}$, the RBF kernel is

(3) $$K\left(x_{i},x_{j}\right)=\exp\left(-\gamma {∥x_{i}-x_{j}∥}_{2}^{2}\right),$$

where γ controls how quickly similarity decays with Euclidean distance. The kernel implicitly maps the waveforms into a higher-dimensional space in which a nonlinear boundary can be constructed without computing the mapping explicitly. The regularisation parameter *C* trades off margin width against training errors, and γ controls the locality of the boundary. Balanced class weights reduced bias toward the majority class, and probability estimates were used for the same validation-based threshold selection applied to the neural models.

### 2.5. Training Details

All neural models were implemented in PyTorch v2.7.0 and trained with the AdamW optimiser, using an initial learning rate of $10^{-4}$, a weight decay of $5\times 10^{-4}$, and a cosine-annealing schedule with an initial warmup. Training ran for up to 300 epochs with early stopping on the validation F1 score, retaining the best checkpoint for testing. These settings were applied identically to the 1D CNN, the MLP, and the LSTM, so performance differences reflect architecture rather than training configuration, and the neural models used these fixed hyperparameters rather than a systematic per-model search. The use of a common training configuration for the neural models was intentional and was intended to compare architectural behaviour under controlled optimisation conditions rather than to optimise each architecture independently. The systematic search was instead performed for the SVM, whose regularisation parameter *C* and kernel bandwidth γ were selected by grid search on the same phantom validation partition.

### 2.6. Evaluation Strategy

All models were developed and evaluated under the same protocol. Training and model selection used only the phantom dataset, divided into fixed training and validation partitions, while the porcine dataset was reserved for a single final test. The three neural models were trained under identical conditions, including weighted binary cross-entropy, positive-class oversampling, the same augmentation, and the same optimiser family, and the SVM received the analogous class-imbalance handling through balanced class weights. Every model was evaluated on the same complete porcine test set, so the reported metrics and confusion matrices refer to an identical number of samples.

Because the model outputs a continuous probability through its final sigmoid, a decision threshold τ converts it into a binary prediction, with a segment classified as cavity entry when p≥τ and non-cavity otherwise. The default τ=0.5 is arbitrary and rarely optimal under class imbalance, so the threshold was not fixed in advance. For every model, the value of τ that maximised the validation F1 score was selected, frozen, and applied unchanged to the porcine test set. Selecting the threshold on validation rather than test data is essential for an unbiased estimate, because tuning it on the test set would leak information and inflate performance. The same procedure was applied to all models.

Performance was quantified with the area under the receiver operating characteristic curve (AUROC), the area under the precision–recall curve (AUPRC), the F1 score, and accuracy. The AUROC measures how well a model ranks cavity above non-cavity signals independently of any operating point. The AUPRC is especially informative here because the cavity class is the minority class, and precision–recall analysis is more sensitive than the AUROC under imbalance. F1 served as the primary threshold-dependent metric and accuracy as an additional global measure. Per-class precision, recall, and F1 are also reported for both classes, defined from the confusion matrix counts as

(4) $$\mathrm{Precision}=\frac{\mathrm{TP}}{\mathrm{TP}+\mathrm{FP}},\;\mathrm{Recall}=\frac{\mathrm{TP}}{\mathrm{TP}+\mathrm{FN}},\;F1=\frac{2\;\mathrm{Precision}\cdot \mathrm{Recall}}{\mathrm{Precision}+\mathrm{Recall}},$$

where TP, FP, and FN are the true positives, false positives, and false negatives of the class. The macro-averaged F1 in Table 2 is the unweighted mean of the two class F1 scores. Per-class reporting matters under imbalance because it separates performance on the clinically critical minority class from that on the majority class, which a single aggregate can obscure.

## 3. Results

### 3.1. Classification Performance

Table 2 presents the classification performance of the four architectures. Both the 1D CNN and the RBF kernel SVM achieved strong performance on the porcine test set, while the remaining neural baselines fell substantially behind.

The 1D CNN reached the highest accuracy (0.869), AUROC (0.904), and macro-averaged F1 (0.833) and the RBF kernel SVM the highest AUPRC (0.878), indicating the slightly better ranking of the minority cavity class. Because each model was trained only once, the small differences between the two, for instance 0.004 in the AUROC, lie within the range expected from run-to-run variation and should not be read as a definitive ranking. The two are best regarded as comparably strong, with the 1D CNN selected on practical grounds as discussed below.

The MLP reached an AUROC of 0.706 and an AUPRC of 0.352. Because the cavity class has a prevalence of 0.250, this AUPRC exceeds the baseline only marginally, showing that global nonlinear combinations of the amplitude samples transferred poorly across the domain shift. Its threshold-dependent performance was correspondingly weak, with an accuracy of 0.703 and a macro F1 of 0.525. The bidirectional LSTM was still weaker, with a precision of 0.403 and a macro F1 of 0.401, indicating that it did not learn a balanced decision boundary for this dataset.

**Figure 2:**
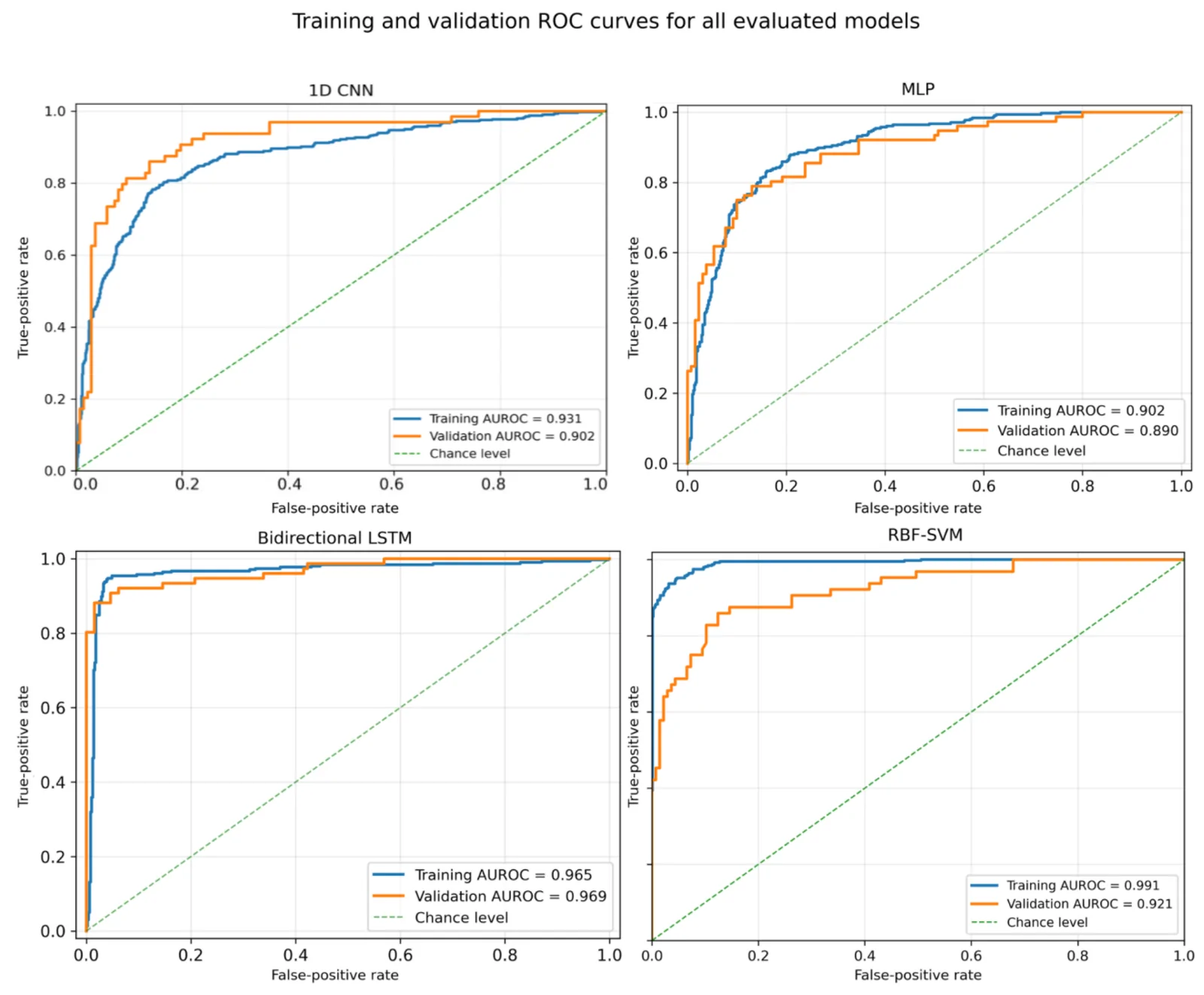
Training and validation ROC curves.

Figure 2 shows the training and validation ROC curves on the fixed phantom partitions. The 1D CNN reached training and validation AUROCs of 0.931 and 0.902, a small gap indicating good discrimination on unseen phantom signals with only modest fitting. The MLP also discriminated well on the phantom, with a validation AUROC of 0.890, but this did not carry over to the porcine set, where its AUROC fell to 0.706.

The bidirectional LSTM attained the highest phantom validation AUROC of all four models, 0.969 against a training value of 0.965, yet the lowest porcine test AUROC, 0.681. This large drop indicates that it fitted characteristics specific to the phantom recordings rather than features that transfer to real tissue. The RBF kernel SVM reached the highest training AUROC (0.991) and a validation AUROC of 0.921. Its larger training–validation gap suggests more fitting to the training data, though its validation discrimination remained strong.

The phantom validation curves therefore do not identify the models that generalise the best. Ranked by the validation AUROC, the LSTM (0.969) precedes the SVM (0.921), the 1D CNN (0.902), and the MLP (0.890), yet on the porcine test set this order is largely inverted. The best-validating model, the LSTM, is the weakest on porcine tissue, while the 1D CNN, only third on validation, is the strongest. Because these curves come from the phantom partitions, they are not expected to match the porcine AUROCs in Table 2. The discrepancy itself is informative since it shows that phantom validation performance poorly predicts performance on real tissue.

Although weighted binary cross-entropy was used to optimise the neural models, epoch-wise training and validation loss histories were not retained during the original experiments. Moreover, the SVM employs a different optimisation objective, preventing a direct comparison of loss trajectories across all four model families. Therefore, the comparative evaluation focused on AUROC, AUPRC, F1 score, and accuracy, which provide a common basis for assessing classification performance.

Table 3 reports per-class precision, recall, and F1 on the test set. The 1D CNN was the most balanced, with a cavity recall of 0.818 and a non-cavity recall of 0.885 and strong non-cavity precision (0.936) and F1 (0.910). The SVM was also strong, with cavity and non-cavity recalls of 0.766 and 0.896. The MLP was far less balanced, combining a high non-cavity recall of 0.877 with a low cavity recall of 0.182 and precision of 0.329, detecting only 28 of the 154 cavity events. The bidirectional LSTM was strongly biased toward the cavity class, reaching a cavity recall of 0.896 at a non-cavity recall of only 0.238. These results confirm that overall accuracy alone does not capture behaviour under class imbalance and that per-class metrics are needed to judge cavity detection against non-cavity rejection.

**Figure 3:**
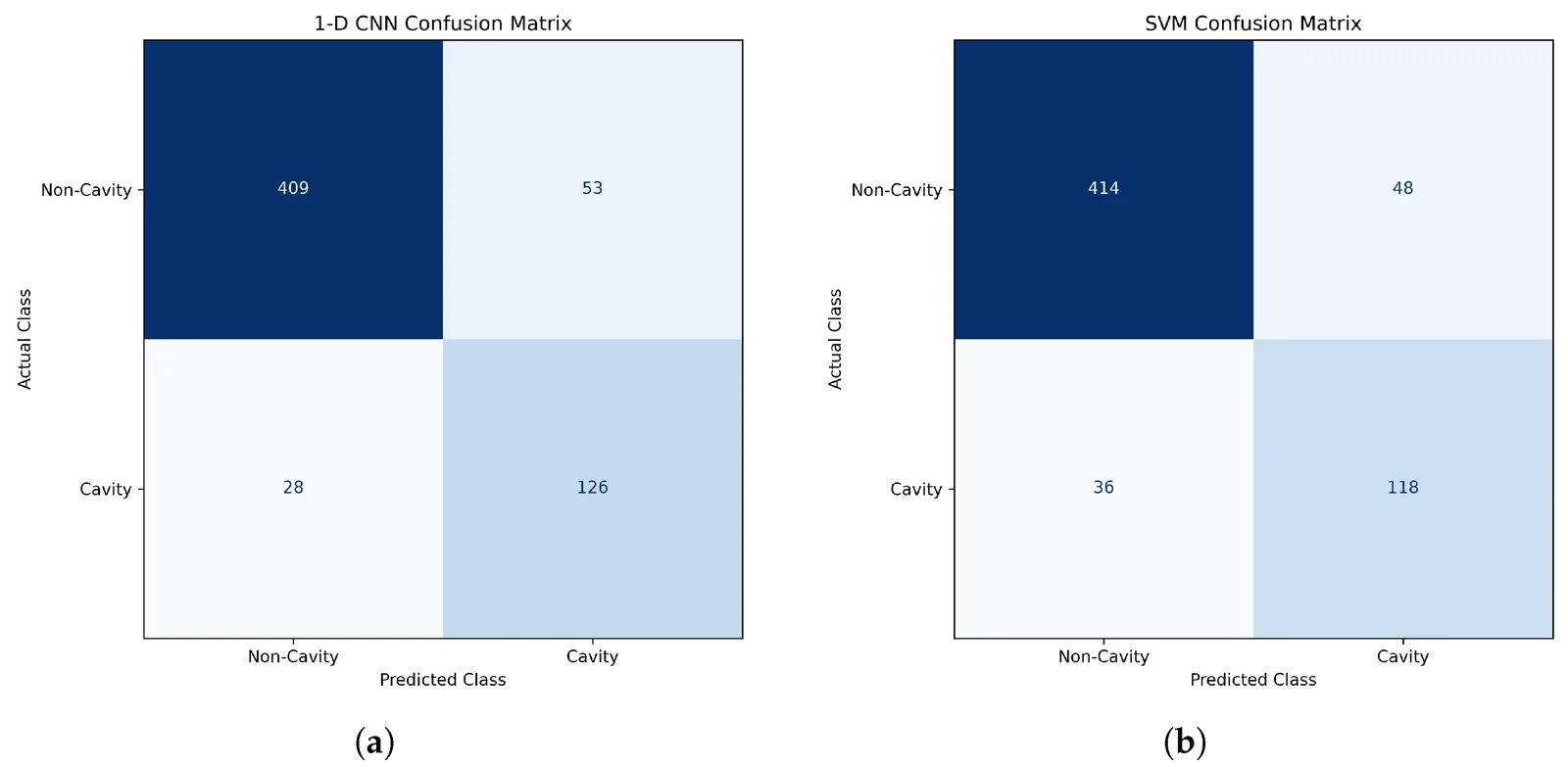
The confusion matrices of the (**a**) 1D CNN and (**b**) RBF kernel SVM on the independent porcine cadaver test set.

The confusion matrices for the 1D CNN and the RBF kernel SVM appear in Figure 3. The 1D CNN classified 126 of 154 cavity and 409 of 462 non-cavity segments correctly, leaving 28 false negatives and 53 false positives. The SVM classified 118 cavity and 414 non-cavity segments correctly, with 36 false negatives and 48 false positives. Both therefore achieved substantially better class balance than the MLP and the LSTM, which produced 126 false negatives and 352 false positives, respectively.

### 3.2. Architecture Selection

The 1D CNN was selected as the primary model. On the porcine test set it reached the highest accuracy (0.869) and AUROC (0.904) with balanced performance across both classes, correctly classifying 409 non-cavity and 126 cavity segments at a cavity recall of 0.818 and precision of 0.704. The SVM achieved slightly higher AUPRC, but the overall difference was small, and the CNN was preferred for several practical reasons. Unlike the SVM, which operates on flattened waveforms, it exploits the temporal structure of the VA signal through learned local filters, making it well suited to the characteristic puncture patterns. It was trained end to end with the augmentation used throughout, produces probabilistic outputs suited to threshold selection, and provides a flexible basis for future extension. While both models captured discriminative information from the VA signal, the convolutional approach offered the best combination of performance, robustness, and extensibility.

The selected 1D CNN was then examined further through a threshold sensitivity analysis and an ablation study, reported below.

#### 3.2.1. Threshold Sensitivity of 1D CNN

The operating characteristics of the 1D CNN were examined at its validation-selected threshold, which matters because a surgical decision-support system needs a clinically defensible sensitivity–specificity trade-off. A lower threshold favours sensitivity to cavity entry; a higher one reduces false positives. Because the threshold was fixed on validation data and then frozen, the values below describe the operating point that would be obtained in practice, without adjustment to the porcine data.

At the validation-selected threshold of τ=0.615, the model reached an accuracy of 0.869, a macro F1 of 0.833, a cavity F1 of 0.757, and a non-cavity F1 of 0.910. For reference, moving to the default τ=0.5 is reported as a separate ablation variant, where accuracy fell to 0.825 while macro F1 rose to 0.846, with AUROC and AUPRC unchanged by construction. The selected point balances the two clinically relevant errors. A cavity recall of 0.818 means most cavity events were detected, while a non-cavity recall of 0.885 means most non-cavity segments were correctly rejected. A missed cavity entry may lead to continued needle advancement, whereas excessive false positives would reduce usability, so the chosen threshold offers a balanced compromise between cavity sensitivity and non-cavity specificity.

#### 3.2.2. Ablation Study of 1D CNN

An ablation study quantified the contribution of the main components of the training and modelling strategy (Table 4). The full configuration is the 1D CNN trained on the VA signals with class-imbalance handling, augmentation, dropout, and validation-based threshold selection. Each variant removed one component while holding the split, input representation, optimiser, and evaluation protocol fixed. The variants removed augmentation, removed class-imbalance handling, replaced the validation threshold with a fixed 0.5, removed dropout, and reduced network depth by removing the dilated convolutions.

The full model reached an accuracy of 0.869, an AUROC of 0.904, an AUPRC of 0.855, and a macro F1 of 0.833. The fixed threshold 0.5 variant differed from the full model only because the validation threshold was 0.615 rather than 0.5, giving an accuracy of 0.825 and a macro F1 of 0.846 at an identical AUROC and AUPRC. Although this fixed threshold produced a marginally higher macro F1 on the test set, it is not the better choice, since selecting it on test data would introduce information leakage. Across the other variants, the AUROC and AUPRC both fell relative to the full model, confirming that augmentation, class-imbalance handling, dropout, and multiscale receptive fields each contribute to ranking performance and generalisation under the domain shift. Accuracy alone can mislead under imbalance. Removing augmentation or dropout lowered accuracy along with the AUROC and AUPRC, yet the no-dropout variant produced a marginally higher macro F1 than the full model. This pattern reinforces the central finding that ranking-based metrics provide the most reliable basis for comparison under the phantom-to-porcine domain shift.

## 4. Discussion

This study evaluated whether Veress needle entry into the abdominal cavity can be identified automatically from the proximally acquired VA signal, comparing four classifier families under a unified protocol. All models were trained, selected, and threshold-tuned on a synthetic phantom and evaluated once on an independent porcine cadaver dataset, so every test result reflects generalisation across a phantom-to-porcine domain shift. The 1D CNN and the RBF kernel SVM clearly outperformed the other baselines. The CNN reached the highest accuracy (0.869) and AUROC (0.904) and the SVM the highest AUPRC (0.878), and both detected the cavity class with high recall while rejecting most non-cavity events. To our knowledge, this is the first demonstration that the peritoneal entry event earlier described qualitatively in porcine cadavers [34] and in a human body donor [35] can be identified by a trained classifier operating on the time-domain VA waveform.

The performance ordering follows from the properties of the four architectures. The peritoneal puncture is a brief, high-energy transient whose discriminative structure is local in time but can occur at different positions within a segment [34,35]. The 1D CNN suits this well, since its shared, translation-tolerant filters detect the same short pattern wherever it occurs and its multiscale receptive field captures both the sharp excitation and its context. The SVM discards temporal ordering by flattening the waveform, but its nonlinear kernel still separates the classes effectively in the high-dimensional sample space. The MLP, which also flattens the waveform but lacks the kernel’s implicit mapping, transferred poorly (porcine AUROC 0.706) and produced an unbalanced operating point, detecting few cavity events (recall 0.182) while over-predicting the non-cavity class. The bidirectional LSTM performed the worst on porcine tissue, remaining strongly biased toward the positive class (cavity recall 0.896 at a non-cavity recall of 0.238). This contrasts with reports from other one-dimensional biomedical domains, where recurrent models outperform convolutional and classical alternatives on ECG data [44]. The difference most likely reflects the input rather than any inherent weakness of recurrent models. ECG beats are quasi-periodic and pre-segmented, so long-range dependencies are informative and learnable, whereas here the decisive information is concentrated in a single brief transient with little for the recurrent state to integrate. In particular, LSTM reached the highest phantom validation AUROC (0.969) yet the lowest porcine AUROC (0.681), fitting phantom-specific characteristics rather than transferable features. The result should therefore be read as a bound on the tested single-layer configuration, not as a general verdict on the recurrent modelling of VA signals.

Measuring performance across a phantom-to-porcine domain shift is central to this work. Training on a controlled, inexpensive, reproducible phantom and testing on biologically variable cadaveric tissue is a demanding test of generalisation and more informative than within-phantom accuracy. The design was also a practical necessity, since the porcine dataset is comparatively small and insufficient on its own to train deep models reliably. The two datasets were deliberately not combined, because pooling different acquisition domains could introduce bias and obscure the very shift the evaluation is meant to measure. The same considerations shape the choice of metrics. With a minority cavity class and differing distributions, accuracy and a single thresholded F1 can mislead, whereas the AUROC and AUPRC characterise ranking independently of the operating point. The ablation results support this, as removing augmentation, class-imbalance handling, dropout, or the multiscale receptive field each lowered the AUROC and AUPRC on the porcine set. The threshold analysis makes the same point. The fixed threshold 0.5 variant gave a marginally higher macro F1 than the validation-selected 0.615 but shares an identical AUROC and AUPRC, and choosing a threshold on the test set would leak information and inflate performance. Fixing the operating point on validation data before testing is therefore essential for an unbiased estimate.

Distinguishing peritoneal entry from other tool–tissue interactions is clinically important, because needle misplacement, whether preperitoneal or in an organ, remains a major source of access complications, and conventional tactile and spring mechanism cues are subjective, especially for less experienced surgeons [9]. An automated indication from the proximally acquired signal is attractive because it is non-invasive, requires no active component inside the patient, and can be added to unmodified standard Veress needles [28,29]. Prior machine learning work on VA needle–tissue signals has targeted tissue type, material properties, or temperature in phantoms and non-biological objects [37,38,39,40,41]. Instead, this study addresses a discrete clinically defined event and benchmarks several model families in a biological test set, complementing earlier descriptive analyses [34,35] by showing that the puncture signature can be captured by a trained classifier rather than by manual, video-anchored annotation.

Several limitations qualify these findings. First, both datasets are ex vivo. Post-mortem tissue lacks perfusion, muscle tone, and respiratory and cardiac motion, all of which may alter needle damping and the puncture transient in living patients [35], so the results show cross-domain feasibility rather than clinical performance. In vivo evaluation, first in a porcine model and then in patients with varying body mass index and abdominal wall thickness and prior surgery, remains necessary. In the porcine cadaver experiments, the Veress needle was also inserted before insufflation, as in the normal surgical procedure, so the influence of intra-abdominal pressure on the VA signal was not part of this study. Whether changes in intra-abdominal pressure alter the VA signal generated during cavity entry should be investigated in future patient studies with clinical data. Second, training used a single phantom source and testing a single porcine dataset, whereas multi-source, multi-subject data are needed to establish a subject-independent threshold. Third, all classifiers operated on the time-domain waveform after only DC offset removal and background noise reduction. Richer time–frequency representations, such as spectrograms or wavelet scalograms, which benefited related work [37,39], were not evaluated and may improve or complement it. Fourth, each configuration was trained once with a fixed split, so no variance estimates or significance tests accompany the reported differences. Fifth, the degree of model-specific optimisation was not identical across model families. The systematic hyperparameter search was performed mainly for the SVM, whereas the neural architectures were evaluated under a common fixed training protocol. The dedicated tuning of the learning rate, regularisation strength, network depth, hidden dimensionality, and other architecture-specific parameters could therefore change both the absolute and relative performance. The reported comparison should consequently be interpreted as a comparison of the evaluated configurations rather than the maximum achievable performance of each model family. This limitation may be particularly relevant to the bidirectional LSTM, which may have been under-tuned in the tested configuration. Hybrid convolutional–recurrent designs were also not examined. Finally, inference latency and computational cost were not measured on target hardware. The lightweight low-cost convolutional design makes the 1D CNN suitable in principle for a future real-time implementation, in line with comparable 1D CNN deployments [45,47], but this remains to be demonstrated on the target system.

These limitations point to clear next steps. Richer signal representations, in particular time–frequency features, should be compared with the time-domain waveform. A real-time implementation should be developed and its inference latency benchmarked on the target embedded hardware. The approach should then be validated on progressively more realistic and ultimately in vivo data, from a porcine model to patients. Combining the classifier with the characteristic puncture-phase structure described previously [34,35] may further improve robustness and interpretability.

## 5. Conclusions

This study showed that Veress needle entry into the abdominal cavity can be detected automatically from the proximally acquired VA waveform. Trained and tuned on a synthetic phantom and tested once on an independent porcine cadaver dataset, the 1D CNN and the RBF kernel SVM generalised well across the phantom-to-porcine domain shift, while the MLP and the bidirectional LSTM did not. The 1D CNN was selected as the primary model, reaching the highest accuracy (0.869) and AUROC (0.904) with balanced performance across both classes. To our knowledge, this is the first automated classification of the peritoneal entry event from VA needle–tissue signals, extending the descriptive porcine cadaver and human body donor findings toward an objective, surgeon-independent indication of peritoneal entry that is compatible with standard Veress needles and existing workflows. The main next step is to move toward time–frequency representations and a real-time implementation, validated on progressively more realistic and ultimately in vivo patient data.

## Figures and Tables

**Table 1:** The structure of the phantom and porcine cadaver datasets. The non-cavity class of the phantom dataset comprises 362 fascial resistance events and 254 artefacts.

Dataset	Use	Cavity	Non-Cavity	Total
Phantom	Training	304	486	790
	Validation	76	130	206
	Total	380	616	996
Porcine cadaver	Test	154	462	616

**Table 2:** Performance comparison on the test set. The best value in each metric column is shown in bold.

Model	Accuracy	AUROC	AUPRC	Macro F1	Threshold
1D CNN	**0.869**	**0.904**	0.855	**0.833**	0.615
MLP	0.703	0.706	0.352	0.525	0.710
Bidirectional LSTM	0.403	0.681	0.358	0.401	0.750
SVM, RBF	0.864	0.900	**0.878**	0.823	0.470

**Table 3:** Per-class precision, recall, and F1 score on the test set, evaluated at each model’s validation-selected operating threshold.

Model	Class	Precision	Recall	F1
1D CNN	Cavity	0.704	0.818	0.757
	Non-cavity	0.936	0.885	0.910
MLP	Cavity	0.329	0.182	0.234
	Non-cavity	0.763	0.877	0.816
Bidirectional LSTM	Cavity	0.282	0.896	0.429
	Non-cavity	0.873	0.238	0.374
SVM, RBF	Cavity	0.711	0.766	0.738
	Non-cavity	0.920	0.896	0.908

**Table 4:** An ablation study for the 1D CNN on the independent porcine cadaver test set.

Model Variant	Accuracy	AUROC	AUPRC	Macro F1
Full 1D CNN	0.869	0.904	0.855	0.833
Without training-time augmentation	0.827	0.783	0.746	0.811
Without class-imbalance handling	0.791	0.742	0.681	0.746
Fixed threshold 0.5	0.825	0.904	0.855	0.846
Without dropout regularisation	0.844	0.801	0.761	0.834
Reduced temporal receptive field	0.812	0.766	0.724	0.793

## Data Availability

The data presented in this study are available on request from the corresponding author due to specific regulations and policies within the organisations involved in this study.
